# Bio-inspired magnetic iron oxide nanoparticles: performance evaluation in cationic and anionic dye remediation

**DOI:** 10.1039/d6ra08763a

**Published:** 2026-11-10

**Authors:** Saurav Kumar Das, Asifur Rahman, Jamil Ahmed, Rumpa Kundu, Md. Abu Rayhan Khan, Mahmoud Al-Gawati, Hamad Albrithen, Shofiur Rahman, Habib Md. Ahsan

**Affiliations:** a Chemistry Discipline, Khulna University Khulna-9208 Bangladesh ahsanhru@chem.ku.ac.bd; b Graduate School of Environmental Studies, Tohoku University 6-6-20 Aoba, Aramaki, Aoba-ku Sendai 980-8579 Japan; c Department of Chemistry, Mississippi State University USA; d King Abdullah Institute for Nanotechnology, King Saud University Riyadh 11451 Saudi Arabia mrahman1@ksu.edu.sa; e Research Chair for Tribology, Surface, and Interface Sciences, Department of Physics and Astronomy, College of Science, King Saud University P. O. BOX 2455 Riyadh 11451 Saudi Arabia

## Abstract

The increasing discharge of synthetic azo dyes into aquatic ecosystems necessitates the development of efficient, low-cost, and sustainable remediation strategies. This study investigates the adsorption capacity of green hydrothermally synthesized magnetic iron oxide (Fe_3_O_4_) nanoparticles (IONPs) for cationic and anionic dyes. Comprehensive characterization confirmed the formation of crystalline Fe_3_O_4_. Microscopic analysis revealed polydispersed spherical particles (≈33 nm) capped with phytochemicals, while Vibrating Sample Magnetometer (VSM) analysis demonstrated superparamagnetic behavior with a saturation magnetization of ≈47 emu g^−1^. The adsorption mechanism of the oppositely charged model dyes, anionic Congo Red (CR) and cationic Methylene Blue (MB), is primarily governed by the surface charge density and pH sensitivity of the IONPs relative to their point of zero charge (pH_PZC_ = 7.5). Surface protonation at low pH facilitates the electrostatic removal of anionic CR. In contrast, deprotonation at high pH enhances the sequestration of cationic MB. Kinetic data for both dyes followed a pseudo-second-order model, while isotherm studies showed that CR follows the Langmuir model (*q*_m_ = 190.11 mg g^−1^) and MB aligns with both Langmuir (*q*_m_ = 181.49 mg g^−1^) and Freundlich models. At the optimized pH, the IONPs' surface promotes a synergy of electrostatic attraction and π–π stacking. Furthermore, the IONPs showed excellent reusability, enabling easy magnetic recovery and structural stability, maintaining high removal efficiency and a consistent Fe–O FTIR signature over multiple adsorption–desorption cycles. Therefore, green-synthesized IONPs provide efficient, magnetically recoverable, pH-responsive dye adsorption, offering a sustainable and high-performance solution for future large-scale industrial wastewater remediation.

## Abstract 2

The pH-sensitive surface charge of iron oxide nanoparticles governs the adsorption of cationic methylene blue and anionic Congo Red. Protonation creates a positive surface, strongly attracts the negative sulfonate groups of Congo Red.

## Graphical abstract

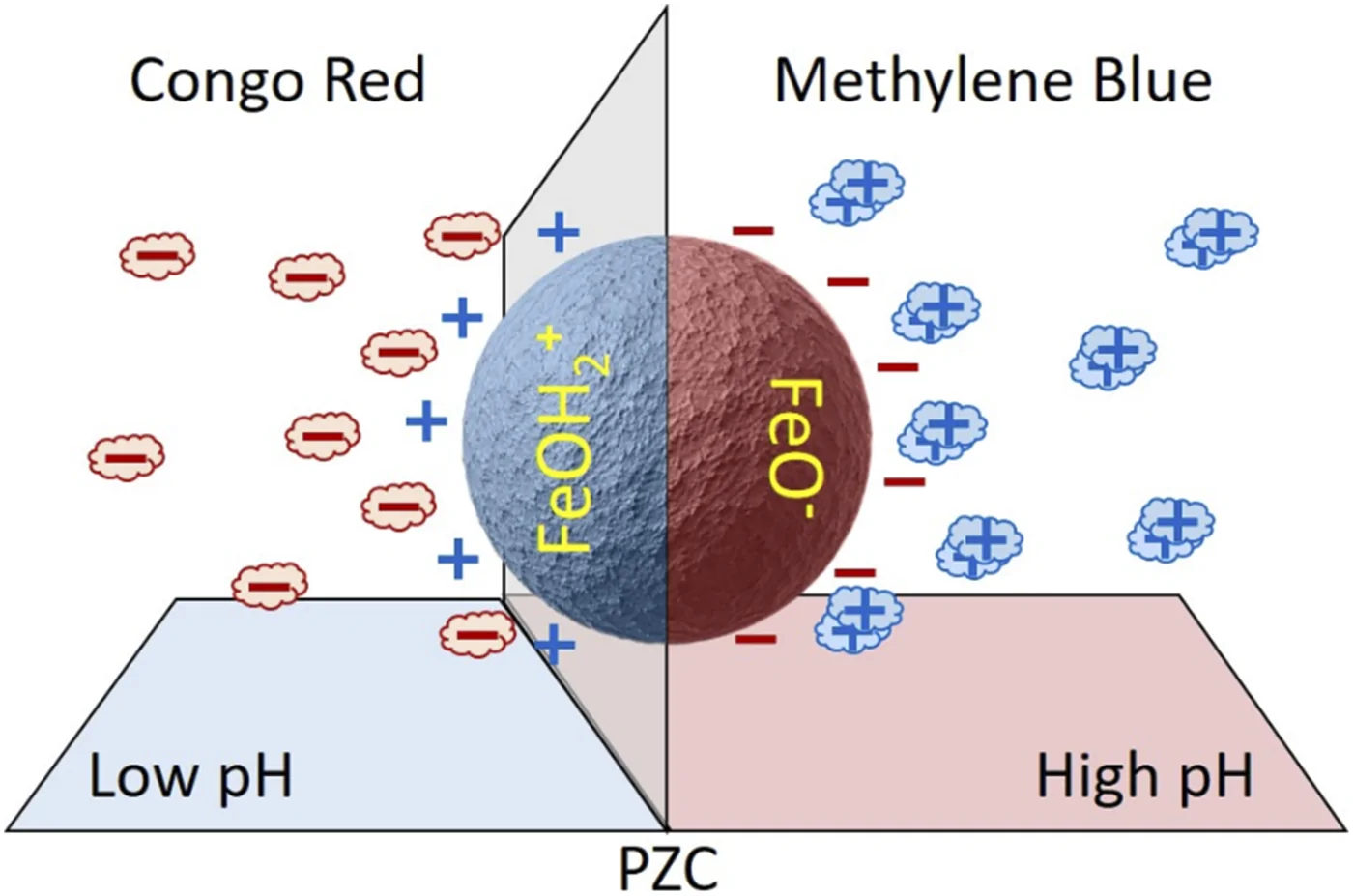

## Introduction

Industrialization has led to a surge in pollutant discharge, posing a significant threat to ecological stability and public health. Therefore, the remediation of industrial wastewater is now a critical priority. Synthetic dyes are ubiquitous across the food, cosmetics, pharmaceuticals, and textile industries.^1,2^ Among these, azo dyes are particularly resilient due to their stable chemical architecture, characterized by aromatic rings and azoic linkages (–N=N–).^3^ This high thermal, optical, and physicochemical stability makes them exceptionally difficult to degrade.^4^ The unregulated release of these compounds into aquatic systems leads to severe environmental consequences, including mutagenicity, carcinogenicity, and skin sensitization.^5^ Furthermore, their presence reduces sunlight penetration in water, disrupting aquatic ecosystems.^6^ Consequently, the remediation of azo dyes from industrial effluents has become a primary focus of contemporary material science and environmental research. To remediate dye-contaminated aqueous solutions, several physical and chemical techniques, including coagulation–flocculation,^7^ oxidation,^8^ membrane separation,^9^ and ultrafiltration,^10^ have been extensively investigated. However, many conventional methods are hindered by significant drawbacks, such as operational inefficiency, high costs, and the generation of carcinogenic secondary pollutants.^11^ While advanced alternatives such as photocatalysis offer superior oxidation potential, they are often limited by high energy consumption.^12^ Therefore, adsorption has emerged as a premier technique due to its operational simplicity, cost-effectiveness, and high removal efficiency without the formation of toxic by-products. While recent research has pivoted toward nanostructured adsorbents to leverage their expansive surface areas, the recovery of non-magnetic nanomaterials remains a significant logistical hurdle.^13^ Without an efficient collection method, these materials risk becoming secondary environmental contaminants themselves. To address this challenge, magnetite nanoparticles, specifically Fe_3_O_4_, have emerged as a transformative solution in wastewater treatment and in broader fields such as biomedicine and materials science.^14,15^ The surging interest in magnetite nanoparticles for environmental remediation is driven by their unique superparamagnetic properties, which enable rapid magnetic separation after use. Beyond their ease of recovery, magnetite nanoparticles offer high surface-to-volume ratios, accelerated reaction kinetics, and a highly modifiable surface, making them a cost-effective and sustainable alternative to traditional, non-magnetic adsorbents.^16^ Thus, the intrinsic magnetic and catalytic properties of iron oxides couple pollutant degradation with simple recovery, illustrating the promise of IONPs for wastewater treatment.

In parallel with their diverse applications, research has intensified to control the morphology and surface chemistry of IONPs. Conventional physical and chemical techniques, such as co-precipitation,^17^ sol–gel,^18^ and thermal decomposition,^19^ effectively produce crystalline nanoparticles. Beyond precursor selection, reaction temperature serves as a decisive factor in governing the synthesis of metal oxide nanoparticles.^20^ Hydrothermal synthesis has established itself as a premier one-step methodology, facilitating high crystallinity and rigorous control over particle size while bypassing the need for post-synthetic annealing.^21,22^ By modulating temperature and precursor concentrations in stainless steel autoclaves engineered to sustain high-pressure conditions, researchers can precisely tune the structural integrity and morphology of the resulting oxides. Ultimately, when these controlled hydrothermal conditions are combined with biogenic precursors, the resulting green-synthesized IONPs emerge as a superior class of nano-adsorbents produced *via* an eco-friendly method.^15^ These materials effectively combine the physical advantages of superparamagnetism with a chemically rich, functionalized surface, offering a robust and sustainable solution for advanced environmental remediation.

This study introduces a sustainable approach to wastewater remediation by reporting a facile, one-step green hydrothermal synthesis of magnetite Fe_3_O_4_ nanoparticles utilizing date palm sap (*Phoenix dactylifera* L.) as a multifunctional reducing and capping agent. By repurposing this locally abundant agricultural by-product from Bangladesh, the synthesis achieves a dual objective: transforming organic waste into high-value nanomaterials while enabling precise control over nanoparticle morphology and yield. The novelty of this work lies in the synergistic exploitation of the sap's chemical profile to tune the surface charge of the IONPs, making them highly responsive to pH for the targeted electrostatic adsorption of both cationic MB and anionic CR dyes and enabling easy magnetic recovery to prevent secondary nano-pollution. Given the severe health risks associated with these dyes, including neurotoxicity and genetic mutations, this research presents a robust, eco-friendly, and cost-effective magnetic composite that exhibits high adsorption efficiency and excellent reusability across multiple cycles, offering a scalable solution for treating hazardous industrial effluents.

## Experimental

### Materials

All reagents used in this research were of analytical grade and utilized without additional purification. Sodium hydroxide (NaOH) (ACS reagent, ≥97.0%, pellets), hydrochloric acid (HCl) (37%, for analysis EMSURE® ACS, ISO, Reag. Ph Eur), ferrous sulfate heptahydrate (FeSO_4_·7H_2_O) (ACS reagents, ≥99%), and ferric chloride (FeCl_3_) (reagent grade ≥97%), along with MB (dye content, ≥82%) and CR (dye content, ≥85%) dyes, were obtained from Merck India. Deionized water was used for all experimental preparations and solutions.

### Date palm sap collection

Date palm sap was sourced from the Jashore district of Bangladesh. For this study, sap collected during the daytime, typically considered agricultural waste, was used, as nighttime harvesting is traditionally reserved for high-value products like jaggery (gur). To ensure purity, the raw sap was filtered and subsequently centrifuged to eliminate suspended impurities. The processed samples were stored in a refrigerator at 4 °C to maintain stability for further experimental use.

### Synthesis of IONPs

IONPs were synthesized *via* a modified hydrothermal approach based on previously established protocols.^23^ To begin, ferric chloride (FeCl_3_) and ferrous sulfate heptahydrate (FeSO_4_·7H_2_O) were dissolved in deionized water at a 2 : 1 molar ratio under continuous magnetic stirring until a homogeneous yellow precursor solution was formed. Subsequently, 18 mL of date palm sap was introduced dropwise into the mixture to act as a stabilizing/reducing agent. The solution pH was adjusted to 10.0 through the incremental addition of 1 M NaOH, followed by 30 minutes of vigorous stirring to facilitate uniform nucleation. The resulting suspension was sealed in a Teflon-lined stainless-steel autoclave and maintained at 180 °C for 5 hours. The appearance of a black precipitate confirmed the formation of magnetite (Fe_3_O_4_) nanoparticles. The product was isolated and washed repeatedly with distilled water until a neutral pH was reached, ensuring the removal of unreacted precursors. Finally, the IONPs were vacuum-dried at 80 °C for 24 hours and stored for further characterization.

### Characterization and instrumentation

The optical, chemical, structural, and thermal characteristics of the synthesized materials were comprehensively evaluated using a suite of advanced analytical techniques. Electronic properties and absorption profiles were recorded using a SHIMADZU UV-1900i spectrophotometer across a 200–800 nm range (1 nm resolution), while chemical functional groups were identified *via* a SHIMADZU IRSpirit FT-IR spectrophotometer (Tokyo, Japan) using KBr pellets at 25 °C. Surface chemistry and elemental states were probed with a Thermo Scientific K-Alpha XPS (Czech Republic) equipped with a monochromatic Kα source (1486.68 eV) and an 180° double-focusing hemispherical analyzer. Morphological and nanostructural insights were obtained through Hitachi SU-8000 SEM (10–15 kV) and JEOL TEM-2100F field emission electron microscopy. Crystalline integrity was verified using a Rigaku SmartLab diffractometer with Cu Kα radiation. Finally, magnetic performance was measured at room temperature using a VSM 8604 (Lakeshore Crytronics), and thermal stability was monitored *via* SHIMADZU TGA-50 at a heating rate of 10 °C min^−1^ under a continuous nitrogen atmosphere.

### Point zero charge determination

The point of zero charge (pH_PZC_) is defined as the specific pH value at which the net surface charge of the adsorbent becomes zero. In this study, the pH_PZC_ of the IONPs was determined using the pH drift method. To establish the background electrolyte, the pH of a 0.01 M NaCl solution was adjusted to various initial values ranging from 2 to 10 using 0.5 M HCl or 0.5 M NaOH. Subsequently, 0.1 g of the IONPs was added to 20 mL of each pH-adjusted solution in capped vials and allowed to equilibrate for 24 hours. After the equilibration period, the final pH of each suspension was measured. The pH_PZC_ was identified by plotting the final pH against the initial pH; the point where the resulting curve intersects the line of equality (pH_initial_ = pH_final_) represents the zero-charge point of the material.

### Adsorption study

To evaluate the adsorption performance of the synthesized IONPs, batch experiments were conducted at room temperature to investigate the effects of pH, contact time, initial dye concentration, and adsorbent dosage on the removal of MB and CR. In a typical optimization run, 10 mg of IONPs were added to 100 mL of a 15 mg L^−1^ dye solution and agitated at 200 rpm for 120 minutes across a pH range of 2 to 12. Following equilibration, the adsorbent was efficiently recovered from the solution using an external magnetic field within 5 minutes, and the residual concentrations of MB and CR were determined by optical absorption at 664 nm and 496 nm, respectively. Each experiment was carried out using three independent replicates. The reported results are the average values, with error bars indicating the standard deviation. We then performed kinetic studies at the optimized pH using 15 mg L^−1^ dye solutions and established adsorption isotherms by varying initial dye concentrations from 5 and 25 mg L^−1^. Furthermore, the effect of adsorbent mass was determined by introducing various doses of IONPs (3–18 mg) into 100 mL of the 15 mg L^−1^ dye solutions. The adsorption capacity, defined as the amount of dye adsorbed per unit mass of adsorbent (*q*_*t*_), was determined using the standard mass balance equation.

The removal efficiency (RE%) was calculated by the following equation:^24^
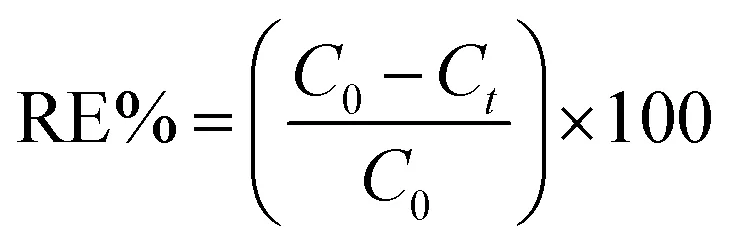


The adsorption capacity (*q*_*t*_, the amount of adsorbed dye per unit mass of adsorbent) of IONPs was calculated by the following equation:^24^
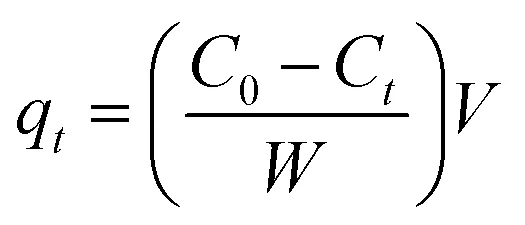
where *C*_0_ and *C*_*t*_ are the concentrations (mg L^−1^) of dye at initial and at time *t*, respectively; *V* represents the volume of the solution (L); *W* is the adsorbent mass (g). *C*_e_ (mg L^−1^) and *q*_e_ denote the equilibrium concentration and equilibrium adsorption capacity, respectively.

### Adsorption–desorption and stability study

To evaluate the recyclability and structural stability of the IONPs, magnetic recovery and regeneration studies were performed at room temperature over five consecutive adsorption–desorption cycles. In each cycle, 20 mg of the IONPs was dispersed into 100 mL of 30 mg L^−1^ aqueous solutions of MB and CR separately, agitated at 200 rpm, and allowed to reach equilibrium for 120 minutes. Leveraging the material's magnetic properties, the adsorbent was efficiently recovered from the solution using an external magnetic field within 5 minutes after reaching equilibrium. The collected nanoparticles were then regenerated by washing with ethanol for 2 hours, followed by thorough rinsing with distilled water to remove any residual solvent or dye. The regenerated IONPs were then reintroduced into the subsequent cycles with identical concentrations of dye solutions and agitation time to assess their sustained removal efficiency. To confirm the chemical stability and structural integrity of the adsorbent throughout the process, FTIR spectra were recorded at three critical stages: in its pristine state (before adsorption), after dye loading (after adsorption), and after regeneration (after desorption).

## Results and discussion

The IONPs were synthesized *via* an eco-friendly hydrothermal method, using date palm sap as both a reducing and a capping agent. Before evaluating their adsorption efficiency, the IONPs underwent rigorous characterization to define their structural and chemical properties.

The UV-Vis absorption spectrum of the date palm-mediated IONPs exhibited a broad, featureless absorbance spanning the entire 200–700 nm range, with a pronounced intensity at 391 nm, as shown in Fig. 1a.^25^ This continuous profile, notably lacking a discrete surface plasmon resonance (SPR) peak, serves as a definitive optical signature of iron oxide formation, distinguishing it from the plasmonic behaviour of noble metal nanoparticles. These spectroscopic features are primarily driven by Fe–O bond excitations and ligand-to-metal charge transfer transitions (notably O^2−^/Fe^3+^) within the oxide lattice. Complementing these data, the visual transition of the reaction mixture from a pale brown to a deep brown-black provided a reliable qualitative confirmation of the synthesis. Such optical characteristics closely mirror established profiles for biosynthesized magnetite Fe_3_O_4_, effectively validating the successful green synthesis of the IONPs.

**Fig. 1:**
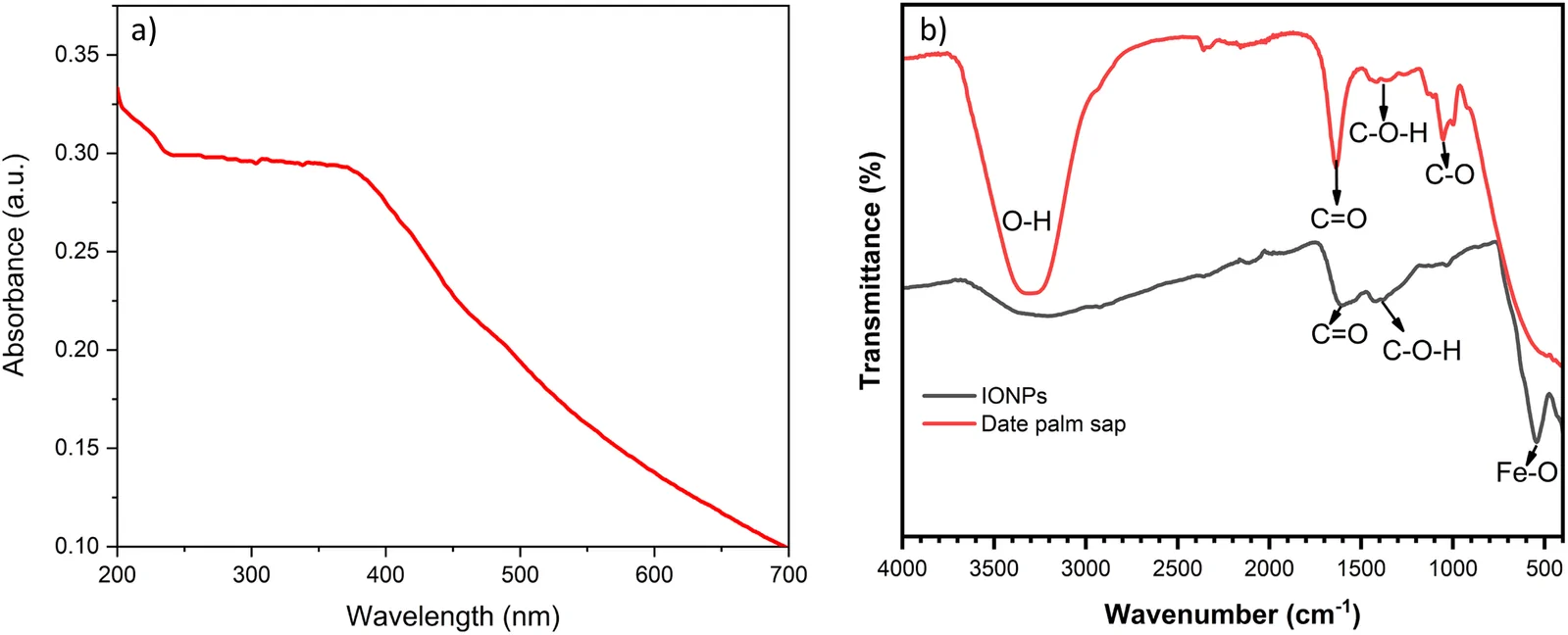
(a) UV-visible spectrum (b) FTIR spectra of the date palm sap and IONPs of IONPs synthesized using date palm sap (condition, 180 °C, 2 hours).

Fourier Transform Infrared (FTIR) spectroscopy highlights the pivotal role of date plum sap phytochemicals in the formation and stabilization of IONPs, as shown in Fig. 1b. The pure sap spectrum exhibits characteristic bands for polyphenols, carboxylic acids, and flavonoids, including a broad O–H stretch at 3300 cm^−1^, a prominent C=O band at 1720 cm^−1^, and various C–O–H and C–O vibrations between 1050 and 1410 cm^−1^.^26–28^ Following the synthesis, these peaks undergo significant attenuation or shifting, specifically the carbonyl and phenolic hydroxyl bands, indicating their direct coordination with iron atoms or their consumption during the reduction of Fe^3+^. Most importantly, the emergence of a new absorption band at approximately 560 cm^−1^, corresponding to Fe–O stretching, confirms the development of the iron oxide core.^17,25,28^ The persistence of residual organic signatures in the IONPs spectrum further demonstrates that these biomolecules serve as capping agents. Ultimately, this confirms that the date plum sap acts in a dual capacity as both a reducing and stabilizing agent, a finding consistent with established literature on green-synthesized iron oxide nanoparticles.

The surface chemistry and elemental oxidation states of the as-synthesized IONPs were analyzed using X-ray Photoelectron Spectroscopy (XPS).

The survey (Fig. 2a) and high-resolution spectra confirmed the presence of Fe, O, and C, with atomic percentages of 10.82%, 39%, 42.74% respectively.^29^ These results validate the formation of the Fe_3_O_4_ core and its subsequent functionalization with date palm sap-derived phytochemicals. The high-resolution Fe 2p spectrum displays two primary peaks at 710.36 eV (Fe 2p_3/2_) and 723.94 eV (Fe 2p_1/2_), shown in Fig. 2b. The spin–orbit splitting of approximately 13.58 eV is a signature characteristic of Fe_3_O_4_. Specifically, the Fe 2p_3/2_ binding energy of 710.3 eV suggests a coexistence of Fe^2+^ and Fe^3+^, pointing toward an Fe_3_O_4_ (magnetite) structure.^30,31^ While the binding energies of Fe_3_O_4_ and γ-Fe_2_O_3_ often overlap, the satellite feature near ∼719 eV serves as a diagnostic tool. The observed suppression of this satellite signal reinforces the presence of mixed-valence Fe_3_O_4_. The spectral profile suggests a predominantly Fe_3_O_4_ core with minor surface oxidation to γ-Fe_2_O_3_, a common occurrence in green synthesis.^32,33^ Deconvolution of the O 1s and C 1s spectra confirms the formation of a robust organic–inorganic interface, where the inorganic iron oxide core is chemically integrated with the date palm sap-derived phytochemicals. Within the O 1s region (Fig. 2c), the peak at 529.56 eV signifies lattice oxygen (O^2−^) within the Fe–O framework, while the signals at 530.93 eV and 532.55 eV correspond to surface hydroxyls/defects and chemisorbed oxygenated species (C–O/C=O), respectively.^34,35^ Complementing this, the C 1s spectrum identifies a dominant peak at 284.8 eV for C–C/C=C bonds, alongside distinct peaks at 286.52 eV (C–O/C–OH) and 288.41 eV (C=O/O–C=O), which represent the polyphenols, sugars, and organic acids acting as capping agents (Fig. 2d).^36,37^ The strong correlation between these oxygenated carbon functionalities and the high-binding-energy oxygen species demonstrates that the extract components are not merely surface contaminants. Instead, they are chemically anchored to the nanoparticle surface. This finding is corroborated by FTIR analysis, which identified similar hydroxyl and carbonyl vibrational bands. The nanoparticles are effectively encapsulated by a stabilizing shell of phytochemicals (polyphenols, organic acids, and sugars). This robust organic–inorganic interface is expected to significantly influence the nanoparticle physicochemical stability and their efficiency in dye adsorption applications.

**Fig. 2:**
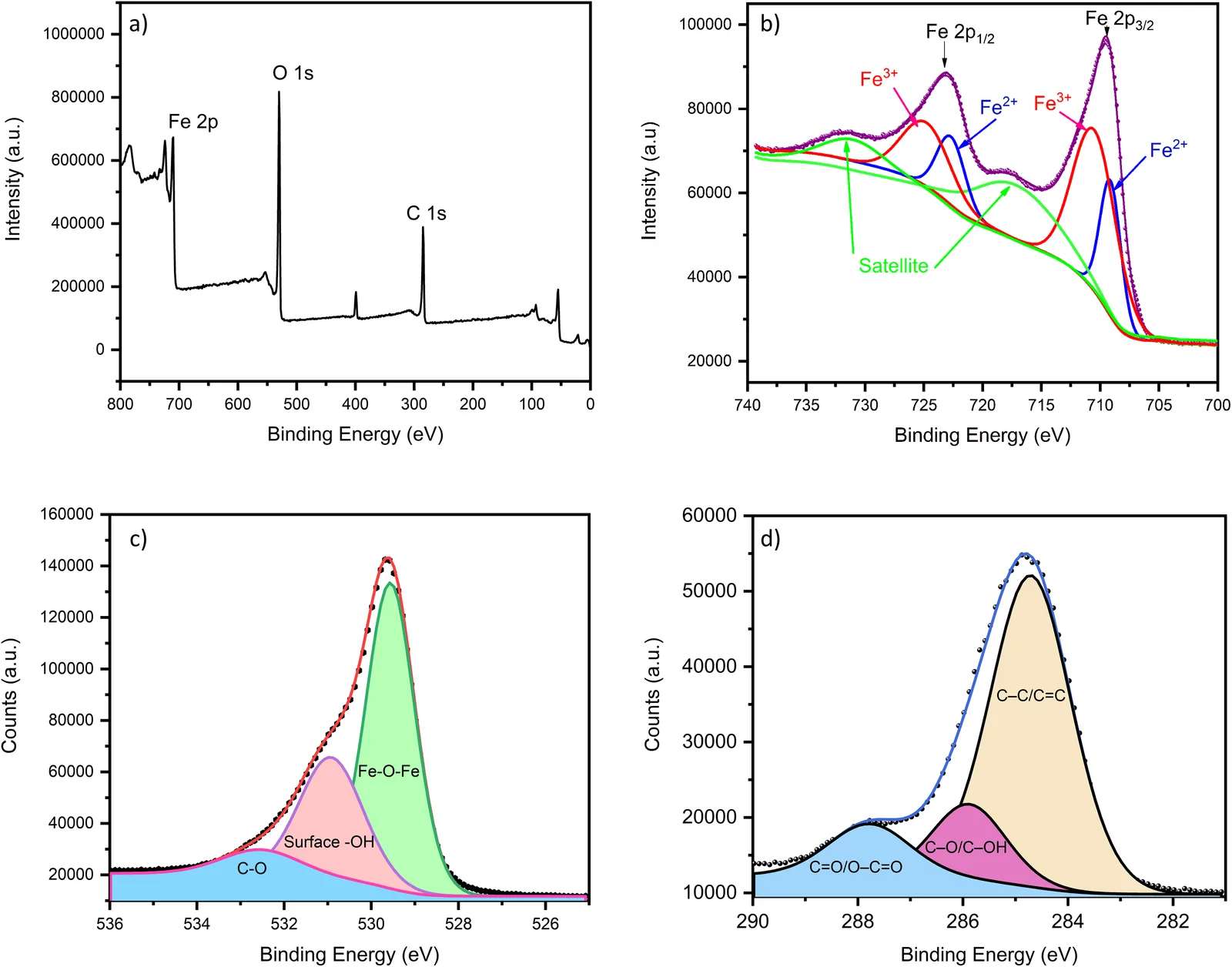
(a) XPS survey spectra of IONPs. (b) XPS spectra of Fe 2p for IONPs, (c) O 1s spectrum of IONPs, and (d) C 1s for the IONPs synthesized using date palm sap (condition, 180 °C, 2 hours).

The XRD analysis of the date palm sap-mediated IONPs confirms a cubic spinel structure, evidenced by distinct Bragg reflections at 2*θ* values of approximately 30.2°, 35.6°, 43.3°, 53.6°, 57.3°, and 62.8° (Fig. 3a). These peaks correspond to the (220), (311), (400), (422), (511), and (440) crystal planes, respectively. These results align closely with standard diffraction data for magnetite (Fe_3_O_4_, JCPDS PDF# 19-0629).^38^

**Fig. 3:**
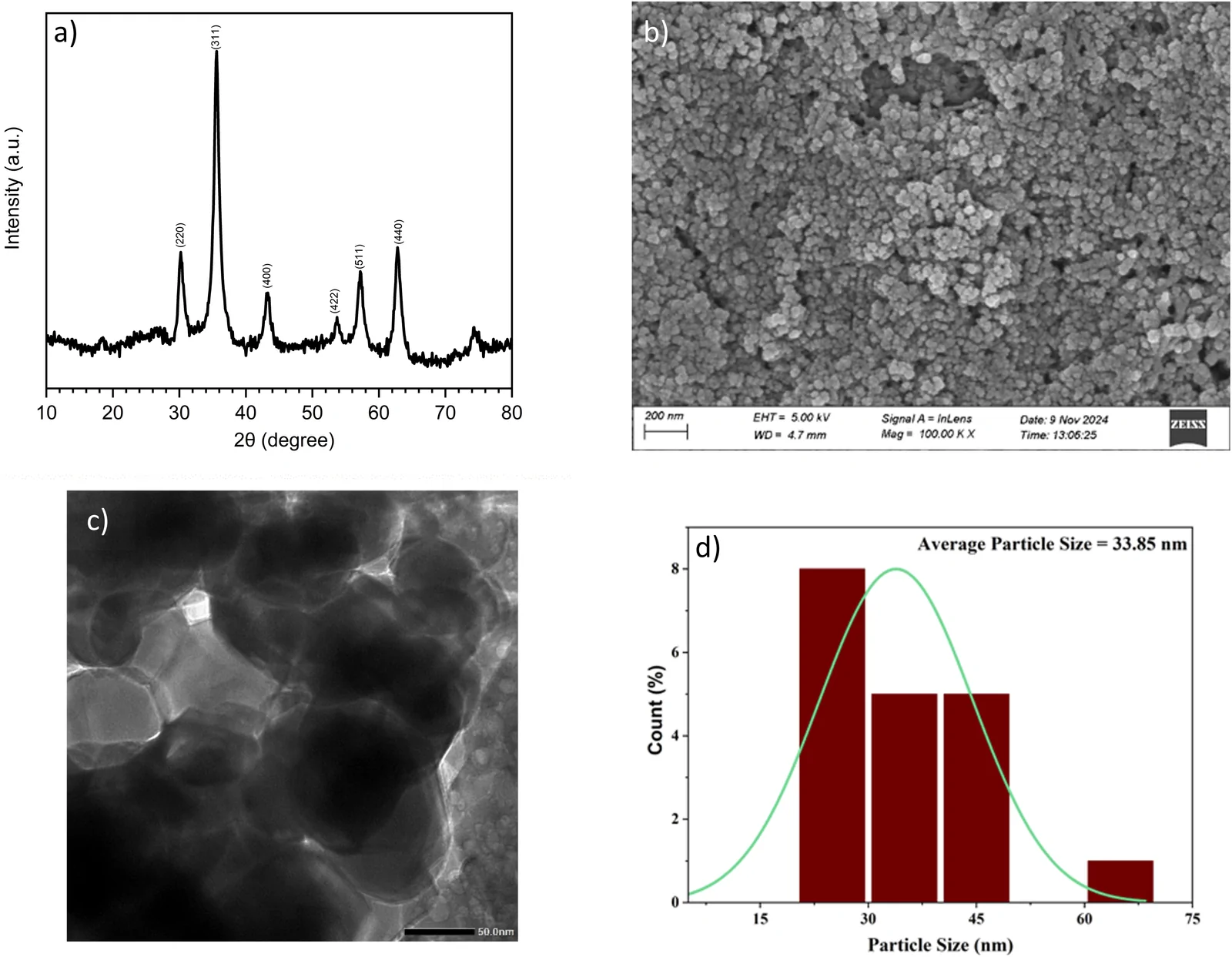
(a) Powder X-ray diffraction pattern of IONPs, (b) field emission scanning electron microscopy (FE-SEM) image of IONPs, (c) Transmission Electron Microscopy (TEM) image and (d) corresponding particle size distribution histogram of IONPs synthesized using date palm sap (condition, 180 °C, 2 hours).

The absence of secondary phases, such as hematite, underscores the high phase purity and crystallinity of the synthesized material. While the structural similarity between magnetite and maghemite makes them difficult to differentiate *via* conventional XRD, the pattern confirms the formation of a spinel iron-oxide phase or a partially oxidized mixture. The average crystallite size (*D*) was calculated using the Debye–Scherrer equation applied to the most intense (311) reflection:^39^*D* = *Kλ*/(*β* cos *θ*)In this formula, *K* represents the Scherrer constant (typically 0.9), *λ* is the X-ray wavelength (1.5406 Å for Cu Kα), *β* is the full width at half maximum (FWHM) in radians, and *θ* is the Bragg angle. The resulting crystallite sizes, using the Scherrer formula on the (311) reflection at 2*θ* ≈ 35.6°, were found to be 10 nm, suggesting that the IONPs are well-crystallized nanoscale ferrites. This nanocrystalline nature often correlates with superparamagnetic behaviour, as the small domain size prevents the formation of permanent magnetic moments at room temperature. From a functional perspective, the small crystallite size and the potential presence of mixed Fe^2+^/Fe^3+^ phases provide a high specific surface area and an abundance of active sites.

Scanning electron microscopy (SEM) analysis revealed that the synthesized IONPs possess a quasi-spherical morphology and form dense, clustered aggregates, as shown in Fig. 3b. Quantitative analysis indicates an ultrafine average particle size of 22 nm. The aggregation observed is likely driven by the magnetic dipole–dipole attractions inherent to magnetite, combined with the relatively mild stabilizing effects of the date palm sap phytochemical coating. While these biomolecules (polyphenols and flavonoids) successfully restricted particle size and facilitated isotropic growth, evidenced by the absence of rod-like or non-spherical forms, the resulting organic layer only partially inhibited the formation of clusters. This plant-derived coating is also reflected in the nanoparticles' non-smooth surface texture, confirming that the extract acts as a functional capping agent that limits uncontrolled growth while maintaining a nanoscale spherical habit.^40^

Transmission Electron Microscope (TEM) analysis of the date palm sap-mediated IONPs (Fig. 3c) reveals quasi-spherical, slightly faceted nanograins with high electron density, a characteristic of dense, crystalline Fe–O lattices that effectively scatter the electron beam. The particles exhibit significant polydispersity and tend to form tight, overlapping clusters, a phenomenon driven by high surface energy, van der Waals forces, and magnetic dipole–dipole interactions that are often exacerbated during sample drying.^41^ While ImageJ analysis yielded an average projected diameter of 33.85 nm (Fig. 3d), this value likely represents clustered aggregates rather than individual primary particles, explaining the discrepancy with the smaller sizes observed in SEM. Unlike SEM, which captures surface-emitted electrons to resolve individual features, TEM provides a two-dimensional projection of the entire particle volume, frequently leading to larger diameter measurements due to 2D overlap and edge-thresholding uncertainties, a common occurrence in the characterization of biogenic nanoparticles.

Thermogravimetric analysis (TGA) of the date palm sap-derived IONPs under a nitrogen atmosphere reveals a three-stage weight-loss profile that confirms the presence of an organic capping layer on a stable inorganic core, as shown in Fig. 4. The first stage, occurring around 150 °C, involves a minor mass drop due to the evaporation of surface-adsorbed moisture and volatile compounds. The most significant weight loss was observed between 200 °C and 450 °C, representing the thermal decomposition and carbonization of plant-derived phytochemicals (polyphenols, proteins, and stabilizers) bound to the nanoparticle surface. Beyond 500 °C, the TGA curve plateaus, demonstrating the high thermal robustness of the Fe_3_O_4_ core, which remains intact even at elevated temperatures.^17^ This characteristic multi-stage pattern, with a stable residual metal-oxide fraction, is a standard hallmark of green-synthesized IONPs and underscores their potential for high-temperature stability and regeneration in environmental applications.

**Fig. 4:**
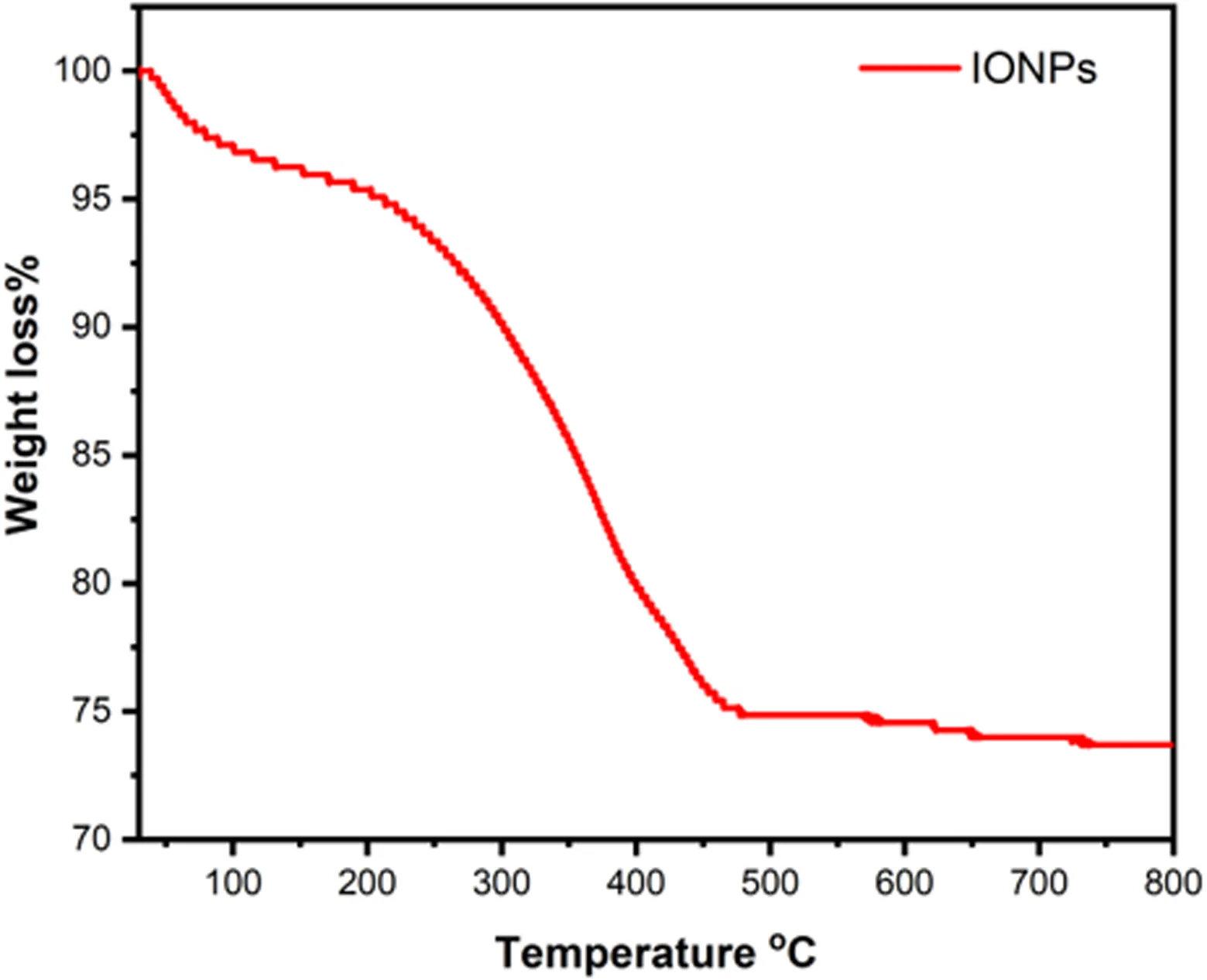
Thermogravimetric analysis (TGA) curve of IONPs synthesized using date palm sap (condition, 180 °C, 2 hours). TGA condition: N_2_ atmosphere and 10 °C min^−1^.

The VSM hysteresis curve of the date palm sap-derived IONPs exhibits a high saturation magnetization (*M*_s_) of ∼47 emu g^−1^, with near-zero coercivity (*H*_c_) and remanence (*M*_r_) as shown in Fig. 5. This classic Langevin-type response confirms superparamagnetic behavior, indicating that the nanoparticles are single-domain structures that magnetize strongly under an external field but lose all magnetic ordering upon its removal. Such properties are ideal for water treatment and catalytic applications, as they allow for rapid magnetic separation and reversible redispersion without the energy loss associated with a hysteresis loop. The *M*_s_ value of these IONPs is superior to several biogenic counterparts, such as those synthesized *via* Piper chaba-mediated nanoparticles (58 emu g^−1^).^17^ This high magnetization suggests a well-crystallized magnetite phase and implies that the phytochemical capping layer does not significantly interfere with the magnetic core. While organic coatings can sometimes create a magnetically dead layer, the date palm sap-derived IONPs maintain a robust magnetic response, making them highly effective for eco-friendly remediation technologies.^42^

**Fig. 5:**
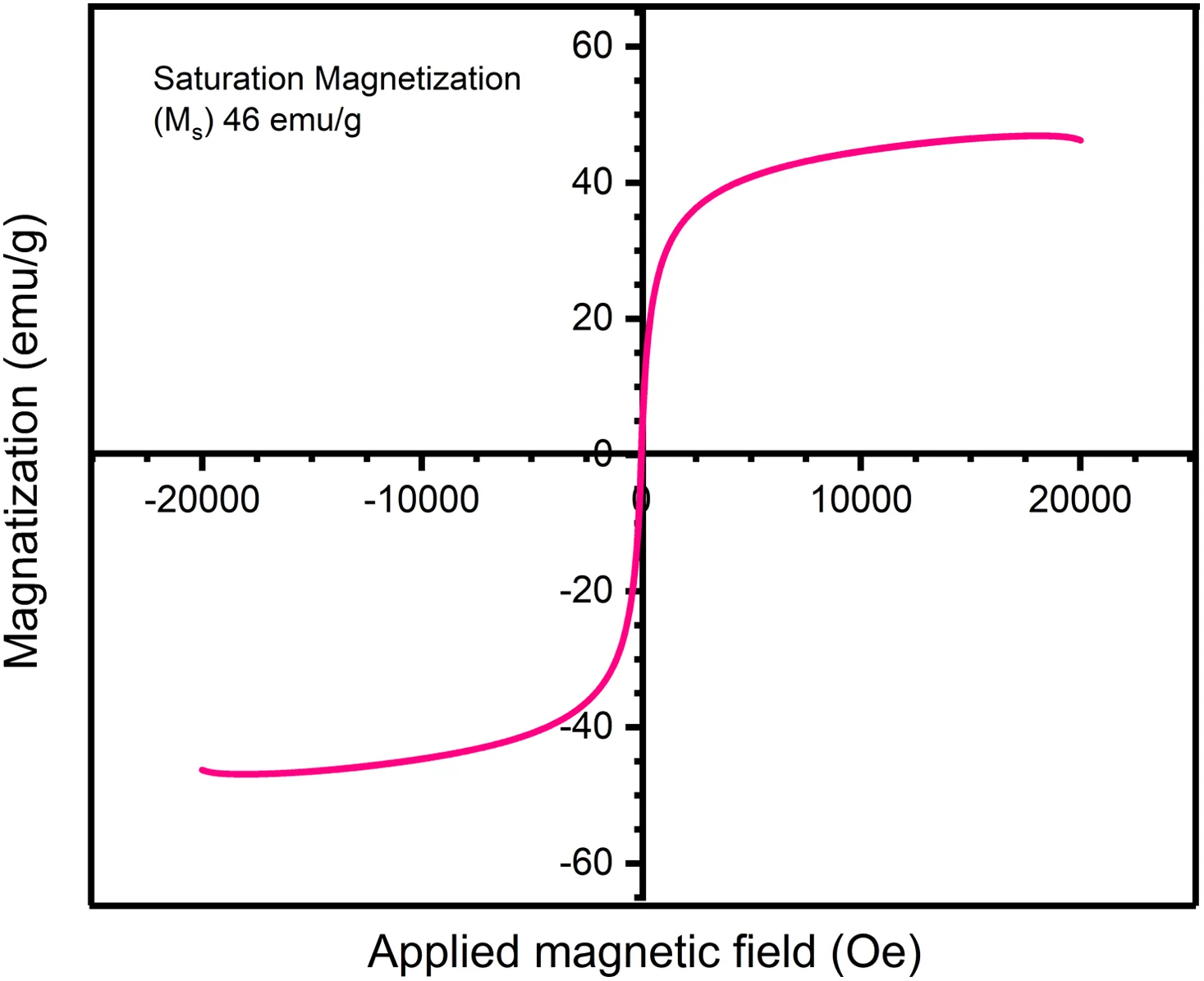
Magnetization curve of IONPs synthesized using date palm sap (condition, 180 °C, 2 hours).

## Adsorption study

### Effect of pH

The point of zero charge (pH_PZC_) for the date palm sap-synthesized IONPs was determined to be approximately 7.5 (Fig. 6a), which serves as the primary determinant of surface–dye interactions.^43^ Below this threshold, surface hydroxyl groups become protonated, forming Fe–OH_2_^+^ and creating a net positive charge.^44,45^ This environment strongly promotes the adsorption of anionic CR, whose negatively charged sulfonate (–SO_3_^−^) groups are strongly attracted to the surface, resulting in ≈100% removal at pH 2 (Fig. 6b).^46^ Conversely, at lower pH, cationic MB is electrostatically repelled, and its uptake is further hindered by H^+^ ions shielding potential active sites. As the pH shifts above the neutral point, the IONPs' surface begins to deprotonate, forming Fe–O^−^ and resulting in a net negative charge.^47,48^ Consequently, CR adsorption diminishes significantly, dropping to approximately 4% at pH 12, while MB adsorption increases sharply (82%) due to the newfound electrostatic attraction. At low pH, very little MB adsorption occurs. These opposing removal trends for CR and MB across the pH scale perfectly mirror the transition of the IONPs' surface charge, confirming that electrostatic forces predominantly govern the adsorption mechanism.^49^

**Fig. 6:**
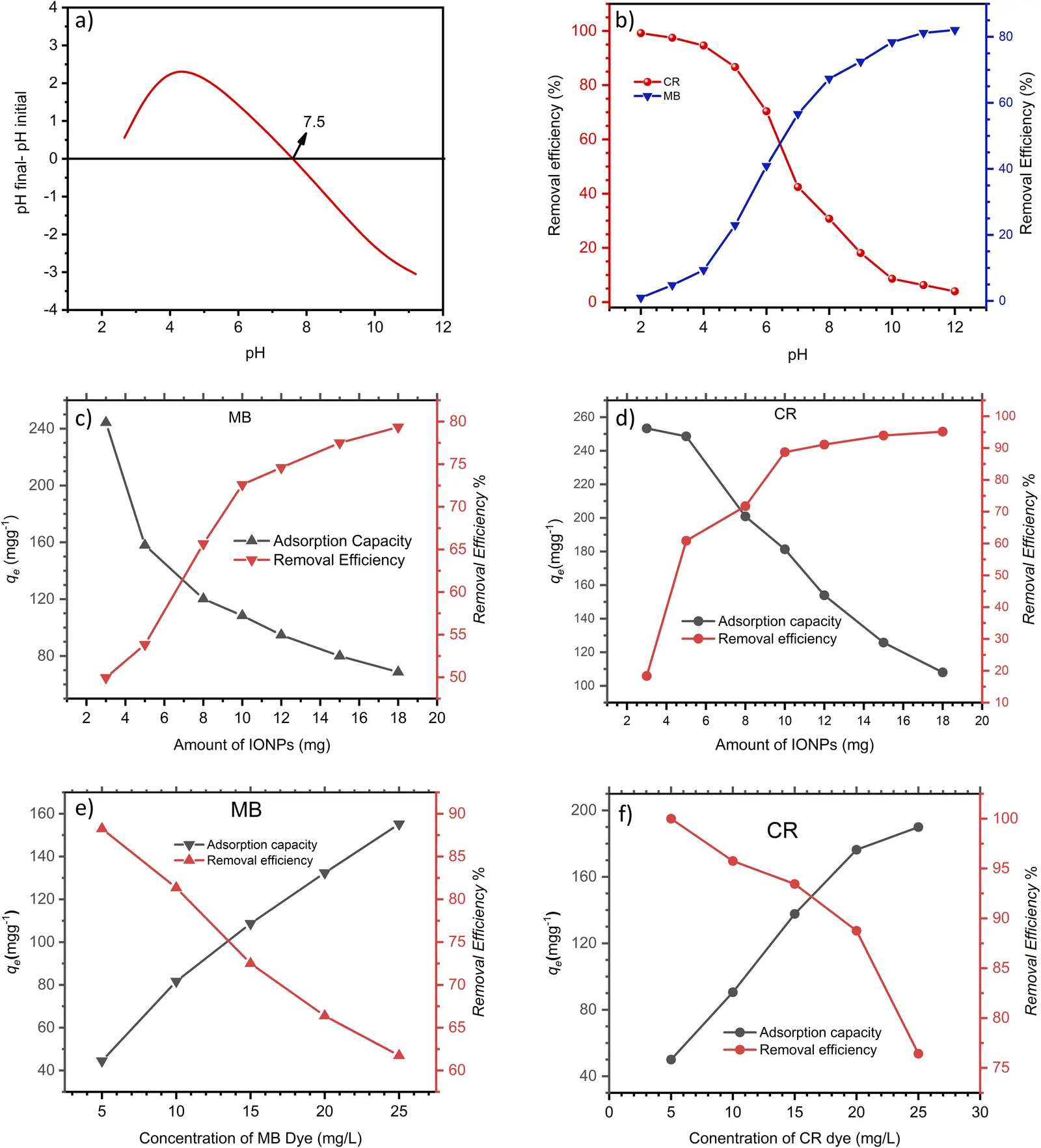
(a) Point of zero charge (pH_PZC_) of the IONPs, (b) effect of solution pH on the adsorption performance of cationic MB and anionic CR on IONPs (condition: 10 mg of IONPs; dye concentration: 15 mg L^−1^; volume: 100 mL, and agitated at 200 rpm for two hours across a pH range of 2 to 12). (c) Effect of dose of IONPs on the adsorption process (condition for cationic MB: IONPs dose: (3–18 mg); volume: 100 mL; MB concentration: 15 mg L^−1^ at pH 10. (d) Effect of dose of IONPs on the adsorption process. Condition for anionic CR: IONPs dose: (3–18 mg); volume: 100 mL; CR concentration: 15 mg L^−1^ at pH 4). (e) The effect of the initial concentration of MB, condition for MB: 5–25 mg L^−1^; volume: 100 mL; IONPs amount: 10 mg and pH: 10. (f) The effect of the initial concentration of CR. Condition for CR: 5–25 mg L^−1^; volume: 100 mL; IONPs amount: 10 mg and pH on the adsorption process.

### Effect of adsorbent dose

The biogenic synthesis of IONPs utilizes date palm sap to encapsulate the metallic core with an organic shell rich in hydroxyl and carboxyl functional groups, which serve as versatile anchors for molecular pollutants. The efficacy of these nanoadsorbents is governed by a sensitive dose–response equilibrium: while augmenting the IONPs concentration provides a larger cumulative surface area and a higher density of active sites, effectively pushing removal efficiencies toward 100%, it simultaneously triggers a precipitous decline in specific adsorption capacity (*q*_e_) (Fig. 6c for MB and Fig. 6d for CR). This phenomenon, frequently documented in nanomaterial research, stems from the underutilization of available sites and the physical agglomeration of particles at high densities, thereby reducing the effective surface-to-volume ratio.^50^

### Effect of initial dye concentration

As the initial concentration (*C*_0_) increases for both CR and MB, the intensified concentration gradient provides a more potent diffusion-controlled driving force that overcomes mass transfer resistance, leading to a higher equilibrium adsorption capacity as active sites are more thoroughly saturated (as shown in Fig. 6e for MB and Fig. 6f for CR).^51,52^ However, because the total number of surface sites remains constant at a fixed dosage, the increase in loading per gram is accompanied by a decline in removal efficiency. The adsorption dynamics are further modulated by the molecular geometry and charge of the adsorbates. The smaller MB molecules with high affinity rapidly populate the nanoparticles surface, whereas the bulkier CR molecules encounter steric hindrance and electrostatic repulsion, resulting in a more gradual approach to a lower adsorption capacity. Eventually, these concentration-dependent trends highlight that while higher dye loads maximize the utilization of the IONPs surface, they necessitate a delicate balance to avoid leaving significant residual pollutants in the effluent.

### Time-dependent adsorption capacity and removal efficiency

The time-dependent adsorption-driven removal process for both dyes, characterized by a rapid initial uptake within the first 20 minutes, followed by a gradual approach to equilibrium, is illustrated in Fig. 7. While MB, a compact cationic dye, exhibits faster kinetics, reaching equilibrium in approximately 60 minutes (*q*_e_ ≈ 108 mg g^−1^) due to its small molecular size and strong electrostatic attraction, the bulkier anionic CR requires roughly 90 minutes to stabilize. However, CR finally achieves a superior adsorption capacity (*q*_e_ ≈ 180 mg g^−1^), as its slower diffusion is eventually offset by a multi-modal binding strategy involving π–π stacking and hydrogen bonding.^53,54^ This higher affinity for CR suggests that the green-synthesized IONP surfaces are functionally enriched with sites that preferentially accommodate anionic species, aligning with literature reports.^55^

**Fig. 7:**
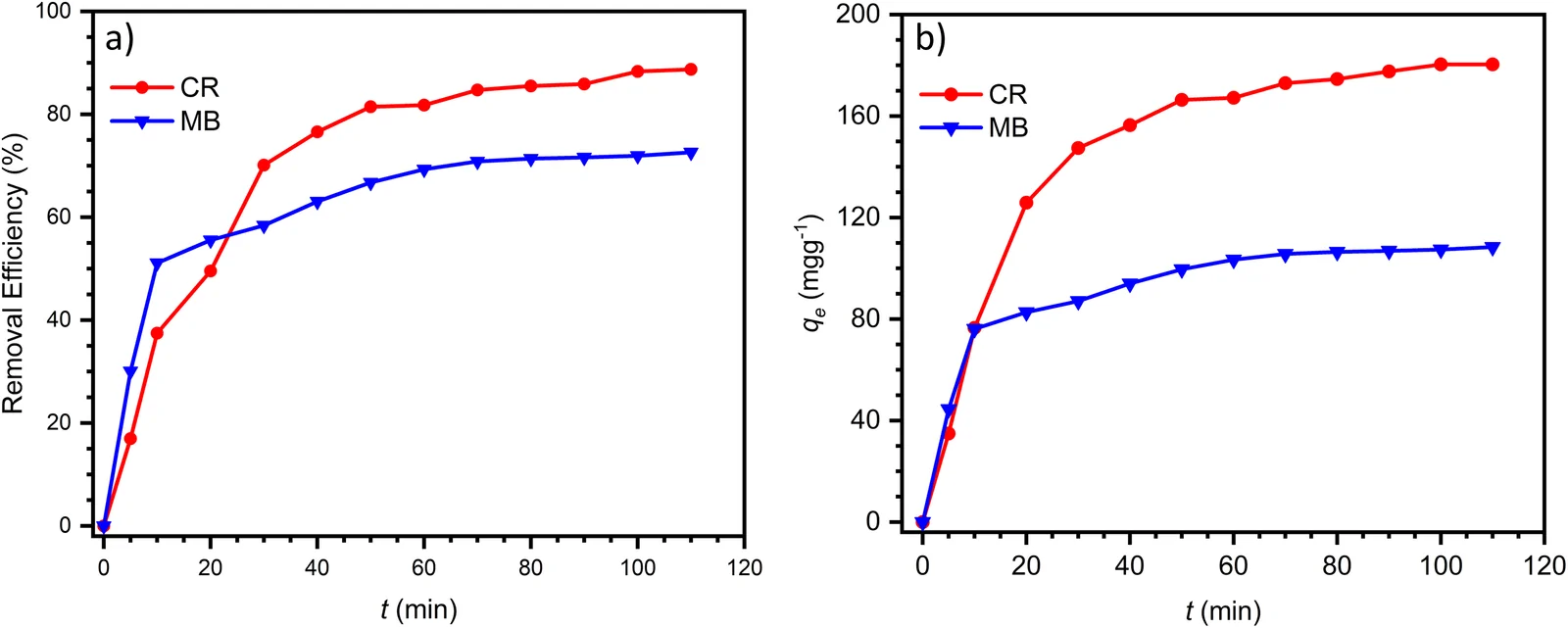
Time dependence of the adsorption of an anionic CR and a cationic MB onto the IONPs. (a) Removal efficiency and (b) absorbance capacity. Condition for CR: dye concentration 15 mg L^−1^; volume: 100 mL; IONPs: 10 mg at pH 4. MB: dye concentration 15 mg L^−1^; volume: 100 mL; IONPs: 10 mg at pH 10.

### Kinetic study

To evaluate the adsorption kinetics of MB and CR onto IONPs, the experimental data were modelled using pseudo-first-order, pseudo-second-order, and intra-particle diffusion frameworks. As shown in Fig. 8, the experimental data points deviate significantly from the pseudo-first-order linear fit (Fig. 8a), whereas the pseudo-second-order plot (Fig. 8b) exhibits a good linear fit for both dyes,^56^ and the obtained parameters of the adsorption kinetics of CR and MB are summarized in Table 1. For MB, the pseudo-second-order model yielded a correlation coefficient *R*^2^ = 0.9937 and a calculated adsorption capacity (*q*^cal^_e_ ≈114.8 mg g^−1^), which closely aligns with the experimental value (*q*^exp^_e_ = 108.4 mg g^−1^). For CR, the pseudo-second-order model provided an excellent fit to the experimental kinetics data (*R*^2^ = 0.9993). Although the calculated adsorption capacity (*q*^cal^_e_ = 205.4 mg g^−1^) was higher than the experimental value (*q*^cal^_e_ = 181.2 mg g^−1^). The deviation may be attributed to experimental uncertainties and the limitations associated with linearized kinetic fitting. However, the higher *R*^2^ value indicates that the pseudo-second-order model provides a satisfactory description of the adsorption kinetics under the investigated conditions.

**Fig. 8:**
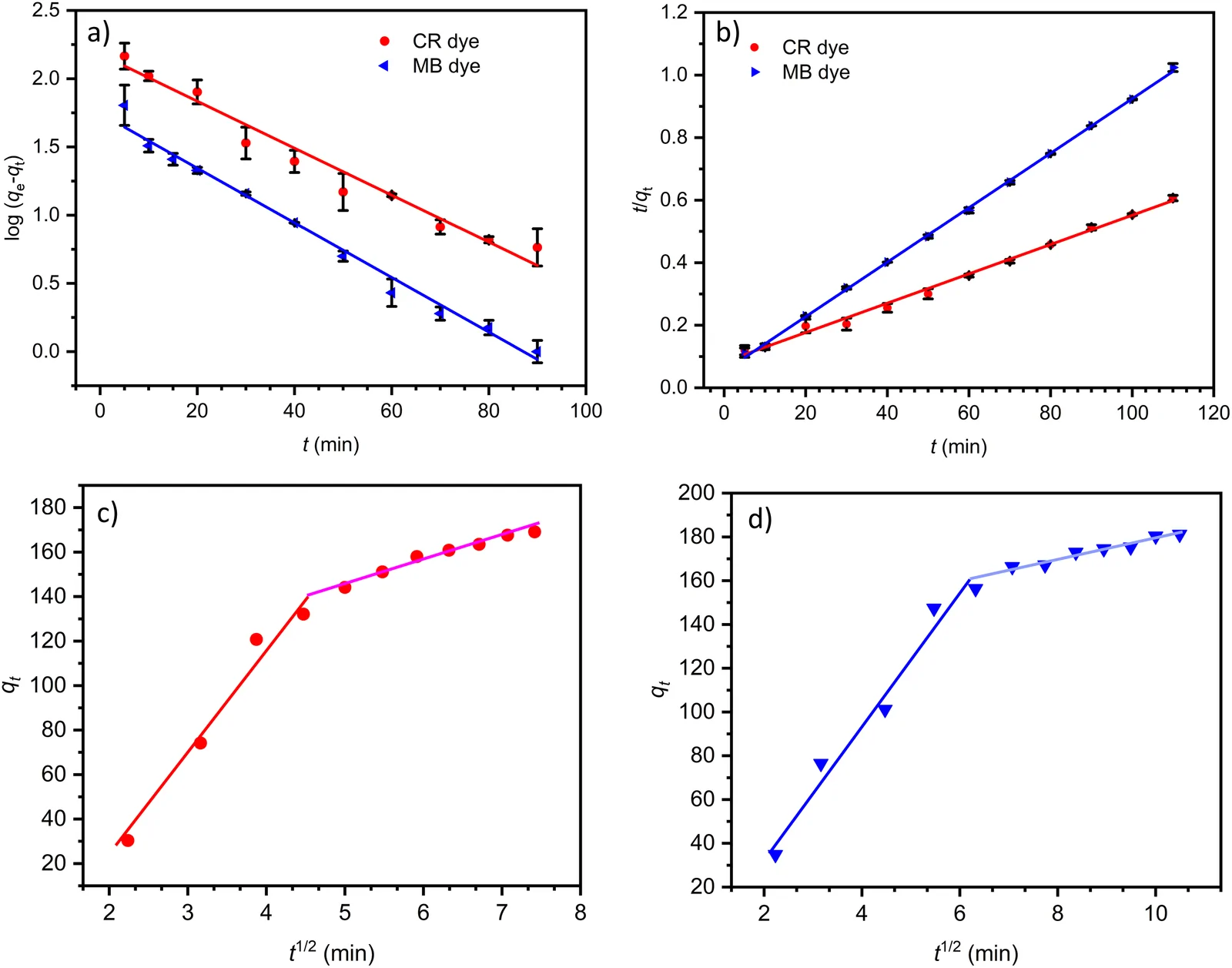
Fitted plot of (a) pseudo-first-order and (b) pseudo-second-order for both dyes. (c) IPD kinetics model for CR and (d) IPD kinetics model for MB.

**Table 1:** Different kinetics models for MB and CR adsorption on IONPs

Models	Parameters	Dye	
		MB	CR
Pseudo-first order	*k*_1_ (×10^−2^/min)	4.9	3.9
	*q* ^cal^_e_ (mg g^−1^)	66.64	142.82
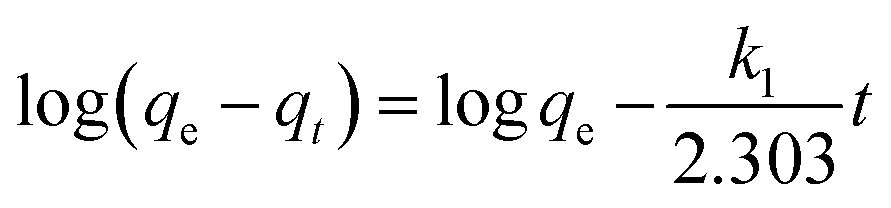	*q* ^exp^_e_ (mg g^−1^)	108.4	181.2
	*R* ^2^	0.9620	0.9850
Pseudo-second order	*k*_2_ (×10^−5^ g mg^−1^ min^−1^)	1.4	2.4
	*q* ^cal^_e_ (mg g^−1^)	114.82	205.4
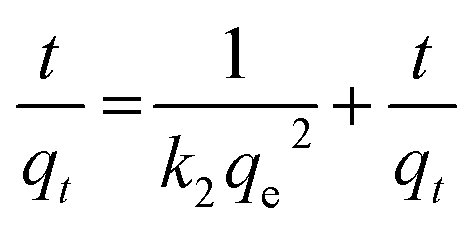	*q* ^exp^_e_ (mg g^−1^)	108.4	181.2
	*R* ^2^	0.9937	0.9993
Intra-particle diffusion	*k*_dif,1_ (mg (g^−1^ min^−1/2^))	47.84	29.90
	*k*_dif,2_ (mg (g^−1^ min^−1/2^))	10.19	4.60
*q*_*t*_ = *k*_dif_*t*^½^ + *C*	*R*_1_^2^	0.9625	0.9633
	*R*_2_^2^	0.9635	0.9495

To investigate the diffusion mechanisms, the Weber–Morris intra-particle diffusion model was employed. Analysis of the plots for MB and CR revealed non-zero intercepts, confirming that intra-particle diffusion is not the sole rate-limiting step in the adsorption process. The transition from a single linear slope to two distinct stages indicates that adsorption is governed by multiple kinetic regimes.^57^ This suggests the simultaneous occurrence of surface adsorption and intra-particle diffusion at varying rates. The initial steep segment represents the rapid migration of dye molecules to the external surface of the IONPs (Fig. 8c for CR and Fig. 8d for MB). The high surface-area-to-volume ratio and the abundance of active sites facilitate this phase. Notably, the negative C values (intercepts) for both dyes suggest that surface mass transfer contributes significantly to the early kinetics.^58^ As surface sites reach saturation, the second stage involves dye molecules penetrating the porous iron oxide shell or organic capping layer. Given the tendency of IONPs to aggregate, this diffusion occurs both within the internal pores of individual particles and through the inter-particle voids within clusters. The diffusion rate constants (*k*_dif_) highlight a significant disparity between the two phases: CR *k*_dif,1_ (stage 1) value is 29.90; *k*_dif,2_ (stage 2) value is 4.60, and MB *k*_dif,1_ (stage 1) value is 47.84; *k*_dif,2_ (stage 2) value is 10.19. The fact that *k*_dif,1_ is substantially higher than *k*_dif,2_ confirms that the surface adsorption phase proceeds much faster than the internal diffusion phase.^58^ This suggests that as the process moves into the interior of the adsorbent, steric hindrance or pore resistance begins to slow the adsorption rate. To better evaluate their performance, the adsorption capacities of the synthesized IONPs for MB and CR dyes were compared with existing literature, as summarized in Table 2.

**Table 2:** Comparison of the maximum adsorption capacities (*q*_e_) of the IONPs for MB and CR with those of other Fe_3_O_4_-based and related magnetic adsorbents

Adsorbent	Synthesis route	Dye	*q*_e_ (mg g^−1^)	Reported conditions/best-fit models	Ref.
Fe_3_O_4_ NPs	Date palm sap, one-step hydrothermal	MB	108.4	Pseudo-second order, Langmuir	This work
		CR	181.2	Pseudo-second order, Langmuir	
Fe_3_O_4_ NPs	*Cordia myxa* leaf extract, co-precipitation	MB	17.79	Langmuir; pseudo-second-order; pH 7.5; 0.50 mg mL^−1^; 60 min	59
Fe_3_O_4_ NPs	*Zanthoxylum armatum* leaf extract	MB	10.47	Langmuir; pseudo-second-order	60
Fe_3_O_4_ NPs	Thermal decomposition	CR	58	Polymolecular (BET-type) isotherm; pseudo-first-order; 25 °C; dye solution pH 5.5	61
Fe_3_O_4_@C	*In situ* solid-phase method	MB	18.52	Exothermic adsorption	62
Fe_3_O_4_/GO	Hydrothermal	MB	135.1	Freundlich; pH 6.0; 50.0 mg adsorbent; 10 min	63
		CR	285.7	Freundlich; pH 6.0; 50.0 mg adsorbent; 10 min	
GO–Fe_3_O_4_	Solution-phase self-assembly	MB	172.6	Equilibrium ≈ 70 min; 87% of initial efficiency retained after 5 runs	64
GO/Fe_3_O_4_	MGO, *in situ* co-precipitation	MB	546.45	Pseudo-second-order; endothermic; effective pH range 5–11	65
		CR	628.93	Pseudo-second-order; endothermic; effective pH range 5–11	
Fe_3_O_4_@MIL-53(Al)	Solvothermal method	MB	70.8	Freundlich; pseudo-second-order	66
		CR	234.4	Langmuir; pseudo-second-order	
Fly ash@Fe_3_O_4_	Fly ash mediated precipitation	CR	154	Freundlich best fit; pseudo-second-order; 10–100 mg L^−1^; equilibrium ≈ 20 min	67

### Isotherm study

The equilibrium adsorption data reveal distinct mechanistic differences between the uptake of CR and MB on the IONPs. The adsorption isotherms of CR and MB onto the surface of IONPs are shown in Fig. 9, and the resulting parameters are presented in Table 3. The adsorption of CR onto the IONPs closely follows Langmuir-type (*R*^2^ = 0.9988) adsorption behavior compared to Freundlich-type adsorption (*R*^2^ = 0.8177). The linear fit of the Langmuir model yields a maximum monolayer adsorption capacity (*q*_m_) of 190.11 mg g^−1^. This suggests that CR molecules adsorbed on a surface exhibiting relatively uniform effective adsorption site behavior. According to the Langmuir theory, once an active site is occupied, no further adsorption can occur at that site,^51,68^ When integrated with pseudo-second-order kinetic data, these results characterize the CR adsorption as a robust chemisorption process. The formation of a single dye layer on the uniform IONP surface is likely driven by electrostatic attraction, interactions between the IONPs surface charges and the anionic dye. Another event is hydrogen bonding, a targeted interaction between the anionic sulfonate (–SO_3_^−^) and amino (–NH_2_) functional groups of the CR molecule.^69–73^

**Fig. 9:**
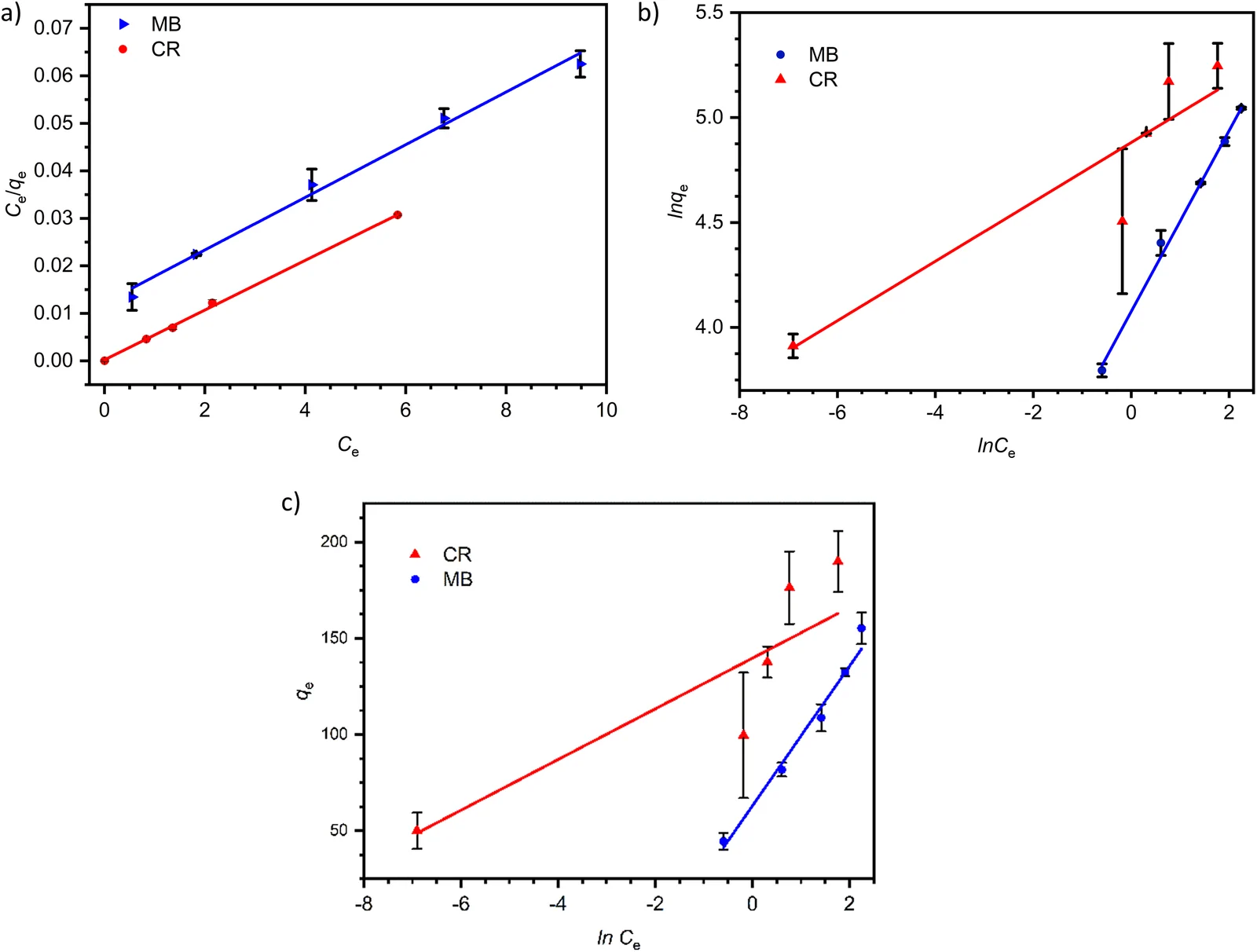
Fitted plots for (a) Langmuir, (b) Freundlich, and (c) Temkin isotherm models for the adsorption of CR and MB dyes on IONPs.

**Table 3:** Comparison of adsorption isotherm constants and statistical fit parameters for CR and MB removal by IONPs

Models	Parameters	Dye	
		MB	CR
Langmuir	*Q*_m_ (mg g^−1^)	181.49	190.11
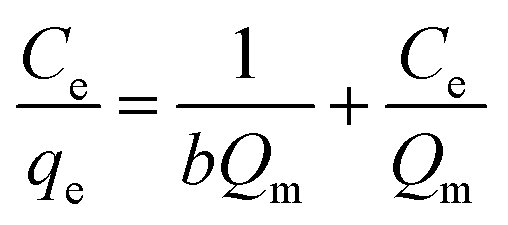	*R*_L_ (*b* = 0.45)		
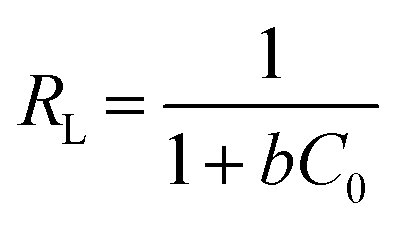	*C*_0_ = 5 (mg L^−1^)	0.3076	0.007
	*C*_0_ = 10 (mg L^−1^)	0.1818	0.0036
	*C*_0_ = 15 (mg L^−1^)	0.1290	0.0024
	*C*_0_ = 20 (mg L^−1^)	0.1000	0.0018
	*C*_0_ = 25 (mg L^−1^)	0.0810	0.0014
	*R* ^2^	0.9331	0.9988
Freundlich	*n*	2.32	6.75
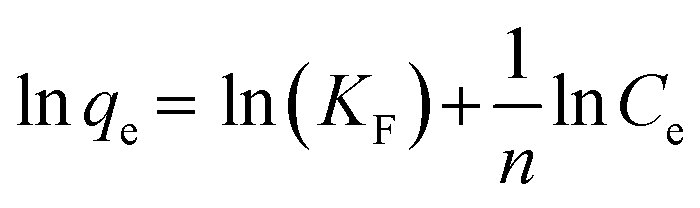	*K*_F_ (mg g^−1^ (L mg^−1^)^1/*n*^)	59.34	130.32
	*R* ^2^	0.9913	0.8177
Temkin	*b*_T_	0.0151	0.0058
*q*_e_ = *B* ln *K*_T_ + *B* ln *C*_e_	*A*_T_ (L g^−1^)	5.28	16.28
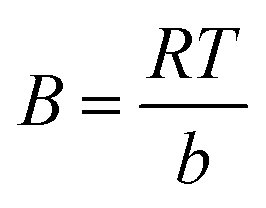	*B* (J mol^−1^)	37.59	14.57
	*R* ^2^	0.9727	0.6548

The adsorption process of MB is predominantly characterized by monolayer coverage on fixed active sites with negligible lateral interactions, as evidenced by the better fit of Langmuir model superior correlation coefficient (*R*^2^ = 0.9931) compared to the Freundlich model (*R*^2^ = 0.9913). Nevertheless, the calculated Freundlich intensity parameter (1/*n* = 0.43) indicates a highly favorable adsorption process and confirms the presence of energetic heterogeneity on the nano-adsorbent surface.^74^ The differences in effective adsorption behaviour can be associated with the variation of interaction between surface functional groups and distinct molecular structure of MB dye.^75^ The Langmuir model, showing a strong correlation with a significant adsorption capacity of 181.49 mg g^−1^, suggests that the IONPs still promotes characteristics of monolayer coverage. The separation factor (*R*_L_), as low as 0.081, confirms that this interaction is highly favourable even as the nanoparticles become saturated.^76^ This robust fit to both isotherm models is driven by the distribution of surface functional groups and dye's molecular structure across the 5–25 mg L^−1^ concentration range. Moreover, it is evidenced that when the surface sites are saturated, the planar aromatic structure of MB enables π–π stacking between adsorbed and free molecules.^61,65^ This interaction triggers a shift from monolayer coverage to a heterogeneous, multilayered distribution, thus satisfying the Freundlich model parameters.^68^

### Proposed dye adsorption mechanism

The adsorption mechanism of ionic dyes onto IONPs is primarily governed by the surface charge density, which is sensitive to the solution's pH_PZC_. The schematic representation is shown in Fig. 10. At this neutral threshold, adsorption for both cationic and anionic dyes is minimized due to the lack of a strong electrostatic drive. However, by adjusting the pH, the surface can be selectively activated for some a specific adsorbate. Below the pH_PZC_, the surface hydroxyl groups undergo protonation (Fe–OH_2_^+^), creating a positively charged surface environment that strongly attracts the negatively charged sulfonate groups (–SO_3_^−^) of CR.^44–46^ This electrostatic interaction is augmented by hydrogen bonding between the amine groups (–NH_2_) of the dye and residual biosynthetic phytochemicals, such as phenolic or carboxylic groups, on the nanoparticle surface.^47^ Consequently, CR removal follows a chemisorption-dominated pathway, forming a saturated, homogeneous monolayer consistent with the Langmuir model. In contrast, increasing the pH above the pH_PZC_ deprotonates the surface (Fe–O^−^), imparting a negative charge that facilitates the sequestration of cationic MB.^47,48^ As described previously, MB exhibits high monolayer capacity (*q*_m_ ≈ 181.49 mg g^−1^) and its adsorption behaviour is better described by the Freundlich model. This indicates that MB molecules interact with a heterogeneous surface where various functional groups and structural defects provide a spectrum of energy levels for bonding. Moreover, beyond simple electrostatic attraction, the planar structure of MB enables π–π stacking interactions,^72,77^ allowing for the development of complex, multilayered accumulation. Thus, while CR reaches a definitive single-layer saturation, MB utilizes the IONPs' high surface-area-to-volume ratio to form a more intricate, multilayered distribution across the heterogeneous active sites. While the FTIR and kinetic data strongly support a mechanism, future studies incorporating post-adsorption XPS will be highly beneficial to directly map the localized chemical state changes and definitively confirm the surface complexation models proposed here.

**Fig. 10:**
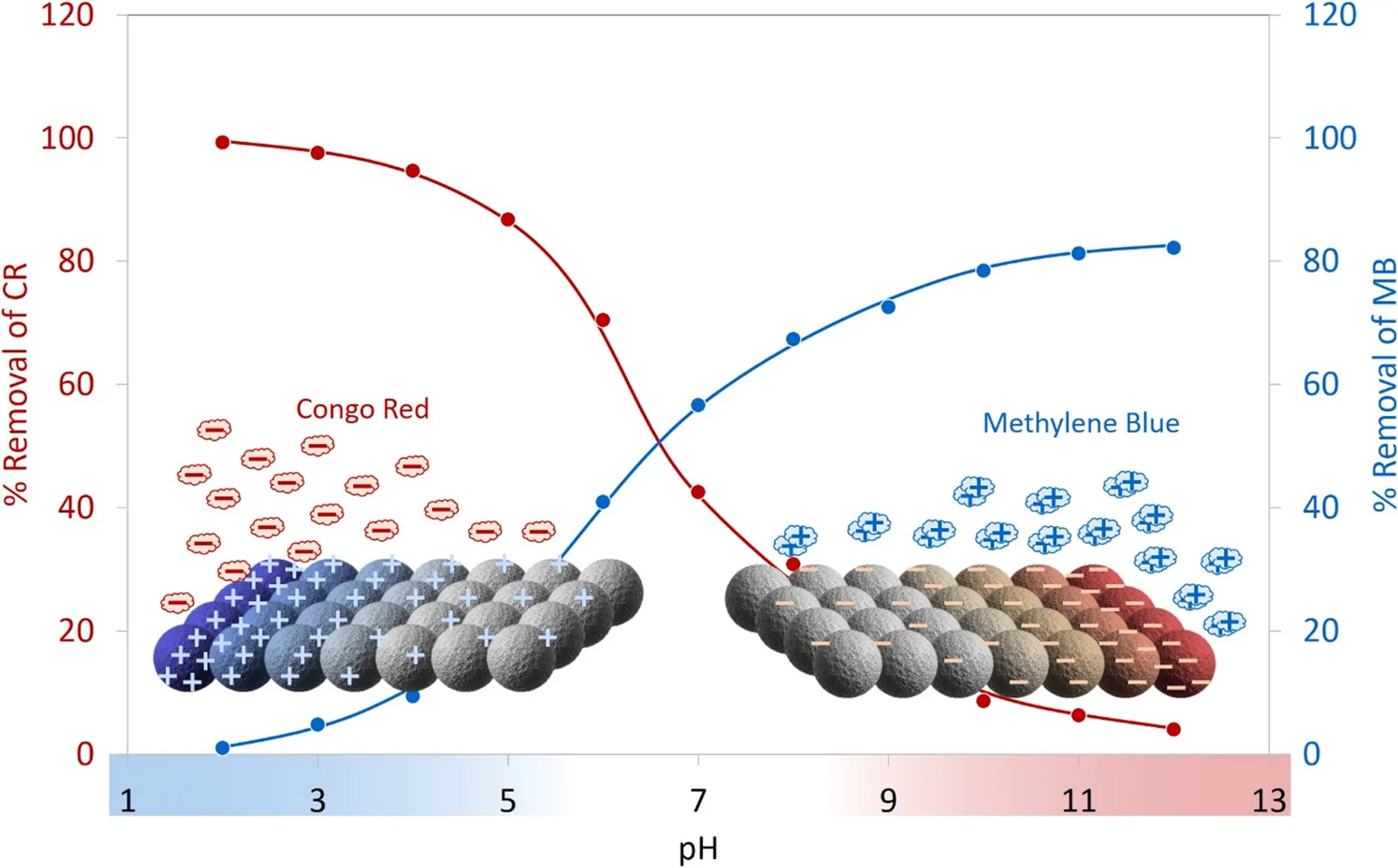
Schematic representation of the pH-dependent proposed adsorption mechanism of anionic CR and cationic MB dyes on IONPs.

### Recyclability and stability

IONPs demonstrate high operational recyclability, although a marginal decline in dye removal is observed over successive cycles; for instance, CR uptake decreased from ≈92% to ≈85% by the fifth cycle, with MB exhibiting a similar downward trend (Fig. 11).

**Fig. 11:**
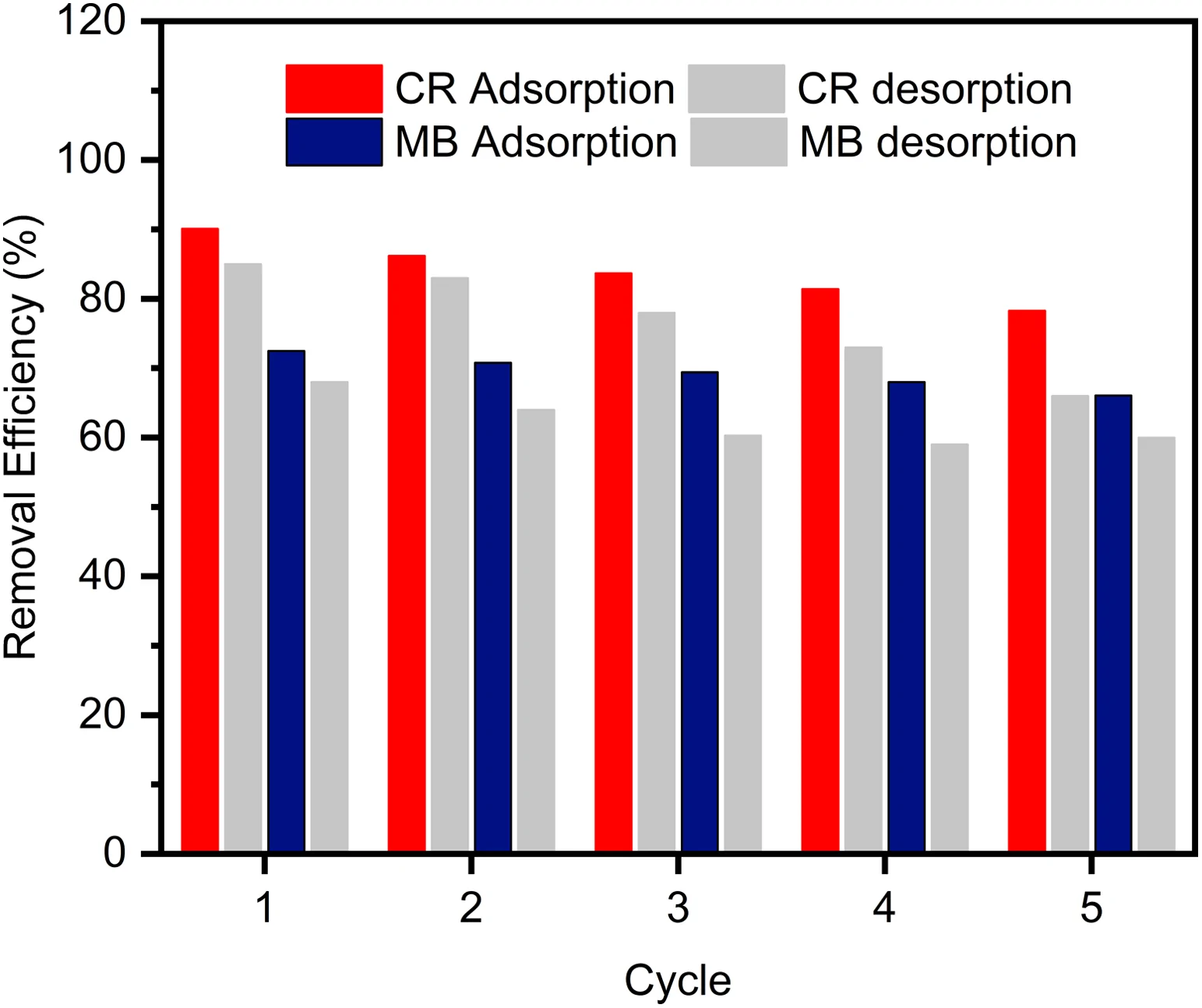
Reusability profile of IONPs for anionic CR and cationic MB adsorption and desorption over five consecutive regeneration cycles.

This performance variance is rooted in their distinct binding mechanisms, where anionic CR forms robust electrostatic or coordination bonds with Fe–OH sites,^44^ while cationic MB adsorption is primarily driven by π–π stacking and attraction to surface hydroxyl groups.^72,77^ Although the progressive loss in efficiency is attributed to incomplete dye desorption or the minor alteration of active sites through leaching and surface damage during regeneration, the retention of 75–85% of the initial capacity after several cycles confirms the inherent durability of the nanoabsorbent for sustainable wastewater treatment. The structural stability of the IONPs was further validated *via* FTIR analysis, which revealed that the characteristic Fe–O peak remained intact after multiple adsorption–desorption cycles (Fig. 12), confirming that the core framework of the nanoabsorbent remains stable and functional for long-term reuse.

**Fig. 12:**
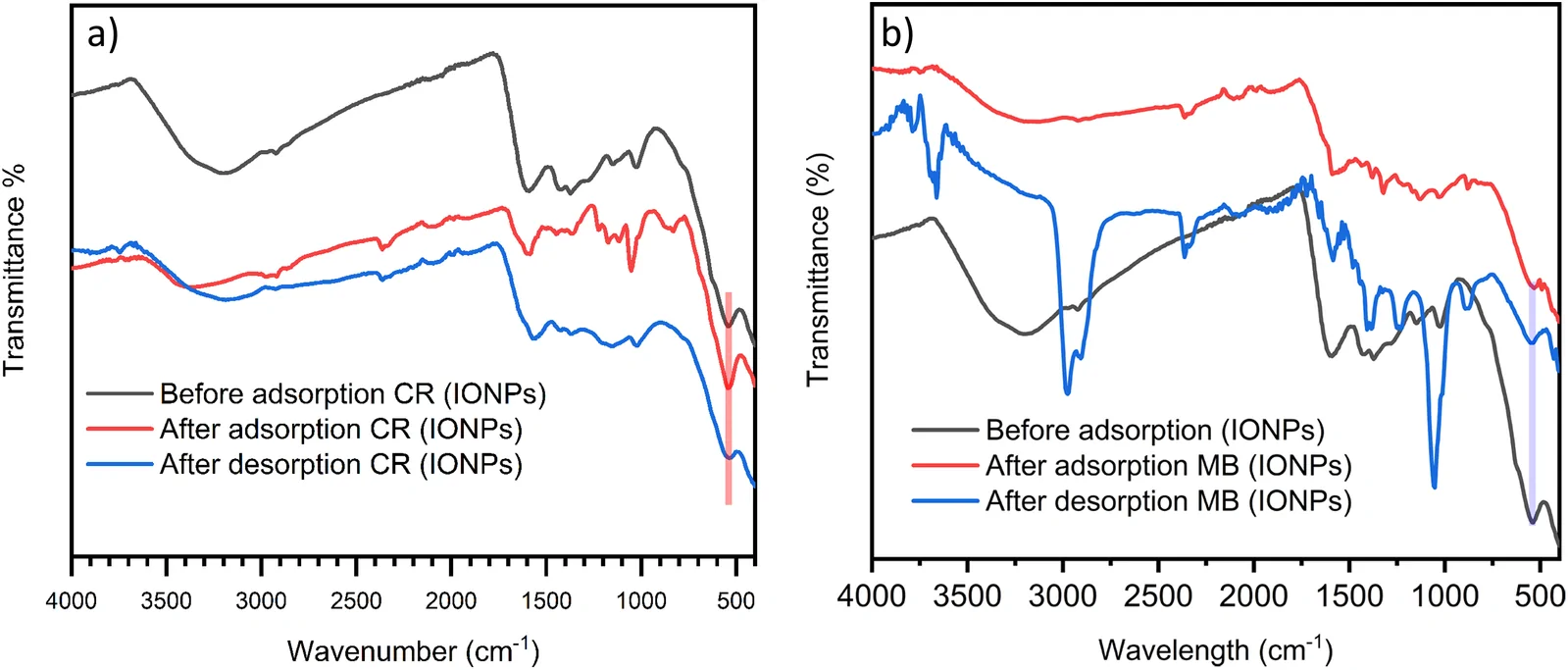
(a) FTIR spectra of IONPs before adsorption, after adsorption of CR dye, and after desorption CR. (b) FTIR spectra of IONPs before adsorption, after adsorption of MB dye and after desorption MB.

## Conclusions

In summary, this study demonstrates that date palm sap is an effective biogenic reducing and capping agent for synthesizing pure spinel-phase magnetite (Fe_3_O_4_) nanocrystals, producing ultrafine, quasi-spherical IONPs naturally capped with phytochemical stabilizers. Comprehensive characterization *via* XRD, XPS, FTIR, and TGA confirmed the formation of a pure iron oxide core functionalized with sap-derived organics, at the same time magnetic testing revealed superparamagnetic behavior with high saturation magnetization (≈47 emu g^−1^) and negligible coercivity. These features enable rapid magnetic recovery and provide a large active surface area for efficient removal of both cationic and anionic dyes. Adsorption results underscored this dual functionality: the nanoparticles rapidly decolorized MB (82% removal, ≈108 mg g^−1^) and CR (≈90% removal, ≈181 mg g^−1^) within 60 to 90 minutes. This versatility is driven by the amphoteric nature of the surface (pH_PZC_ ≈ 7.5), allowing for pH-tuneable attraction of diverse pollutants. Furthermore, the IONPs are still stable after 5 cycles. More importantly, these magnetite nanoparticles achieve dye adsorption while enabling easy magnetic recovery to prevent secondary nano-pollution. By leveraging a sustainable, green synthesis route, this work offers a high-impact, cost-effective solution for wastewater remediation that avoids hazardous chemicals and addresses complex industrial effluents. While the present study establishes a strong fundamental baseline for the high-capacity adsorption of MB and CR by green-synthesized IONPs in controlled aqueous systems, real-world industrial wastewater is highly complex. Future studies will focus on evaluating the competitive effects of co-existing inorganic ions and natural organic matter on the adsorption efficiency, as well as testing the material in continuous fixed-bed column operations to fully realize its industrial application potential.

## Author contributions

Saurav Kumar Das: validation formal analysis and data collection. Asifur Rahman and Jamil Ahmed: writing – original draft and writing – review & editing, Rumpa Kundu and Md. Abu Rayhan Khan: data analysis and methodology. Mahmoud Al-Gawati and Hamad Albrithen: validation, investigation and data analysis. Shofiur Rahman: writing – review & editing, writing – original draft, funding acquisition, validation, investigation, and Habib Md. Ahsan: review & editing, writing – original draft, supervision, funding acquisition methodology, investigation, conceptualization.

## Conflicts of interest

There are no conflicts to declare.

## Data Availability

All the data produced and/or analyzed in the course of this study are presented within this manuscript.
